# Deep Neck Infection in Systemic Lupus Erythematosus Patients: Real-World Evidence

**DOI:** 10.1038/s41598-020-61049-4

**Published:** 2020-03-05

**Authors:** Geng-He Chang, Yi-Cheng Su, Ko-Ming Lin, Chia-Yen Liu, Yao-Hsu Yang, Pey-Jium Chang, Meng-Hung Lin, Chuan-Pin Lee, Cheng-Ming Hsu, Yao-Te Tsai, Ching-Yuan Wu, Ming-Shao Tsai

**Affiliations:** 1 Department of Otolaryngology - Head and Neck Surgery, Chiayi Chang Gung Memorial Hospital, Chiayi, Taiwan; 2 Health Information and Epidemiology Laboratory, Chiayi Chang Gung Memorial Hospital, Chiayi, Taiwan; 3 Graduate Institute of Clinical Medical Sciences, College of Medicine, Chang Gung University, Taoyuan, Taiwan; 4 Department of Medical education, Chiayi Chang Gung Memorial Hospital, Chiayi, Taiwan; 5 Division of Rheumatology, Allergy and Immunology, Chiayi Chang Gung Memorial Hospital, Chiayi, Taiwan; 6 College of Medicine, Chang Gung University, Taoyuan, Taiwan; 7 Department of Traditional Chinese Medicine, Chiayi Chang Gung Memorial Hospital, Chiayi, Taiwan; 8 School of Traditional Chinese Medicine, College of Medicine, Chang Gung University, Taoyuan, Taiwan; 9 Department of Nephrology, Chang-Gung Memorial Hospital, Chiayi, Taiwan

**Keywords:** Systemic lupus erythematosus, Bacterial infection, Prognosis, Risk factors

## Abstract

Systemic lupus erythematosus (SLE) might increase deep neck infection (DNI) risk, but evidence supporting this hypothesis is limited. In this retrospective follow-up study, the SLE–DNI association was investigated using data from the Registry for Catastrophic Illness Patients, which is a subset of the Taiwan National Health Insurance Research Database. All patients newly diagnosed as having SLE in 1997–2011 were identified, and every SLE patient was individually matched to four patients without SLE according to sex, age, and socioeconomic status. The study outcome was DNI occurrence. DNI treatment modalities and prognoses in SLE and non-SLE patients, along with the association of steroid dose with DNI risk, were also studied. In total, 17,426 SLE and 69,704 non-SLE patients were enrolled. Cumulative DNI incidence was significantly higher in the SLE cohort than in the non-SLE cohort (*p* < 0.001). The Cox regression model demonstrated that SLE significantly increased DNI risk (hazard ratio: 4.70; 95% confidence interval: 3.50–6.32, *p* < 0.001). Moreover, in the sensitivity and subgroup analyses, the effect of SLE on DNI was stable. Relatively few SLE–DNI patients received surgical interventions (15.6% vs. 28.6%, *p* = 0.033). The between-group differences in tracheostomy use and hospitalisation duration were nonsignificant. In SLE patients, high steroid doses significantly increased DNI incidence (≥3 vs. <3 mg/day = 2.21% vs. 0.52%, *p* < 0.001). This is the first study demonstrating that SLE increases DNI risk by approximately five times and that high steroid dose increases DNI incidence in SLE patients.

## Introduction

The prevalent infectious disease deep neck infection (DNI) is typically encountered in emergency departments. Patients with DNI typically require intensive care and aggressive treatment. DNI has the likelihood to be life threatening, notably in systemic disease patients and elderly individuals^1–3^. Immunocompromised patients do not show usual symptoms of DNI, making its early diagnosis difficult, possibly increasing complications and mortality^1,3–5^. Therefore, investigating the influence of immunosuppressing diseases on DNI is important.

Systemic lupus erythematosus (SLE) renders its affected patients vulnerable to infection; in addition, in such patients undergoing dialysis, infection is a notable cause of mortality^6–9^. SLE activity itself and long-term usage of immunosuppressants are considered the two main causes for infection susceptibility^6,10^. In their case series of 130 patients with DNI, Yang *et al*.^11^ reported two cases of DNI in patients with SLE under steroid therapy. However, the influence of SLE on DNI was not investigated in depth. In addition, the association between the duration of SLE, steroid dosage, and DNI risk remains unknown. We therefore conducted this real-world study with the primary purpose of probing the influence exerted by SLE on DNI incidence, treatment, and prognosis.

## Methods

### Data source

As of 2018, Taiwan’s well-known National Health Insurance covered approximately 99.6% of the country’s residents^12,13^. The National Health Insurance Research Database (NHIRD) contains the entirety of NHI beneficiaries’ medical claims data; these data include information related to disease diagnoses made at the time of clinical visits and hospitalisation, examinations received, drugs and doses prescribed, payments made, procedures and surgeries received, locations of residence, and income levels—all generated during insurance reimbursement in electronic format^13,14^. Diagnoses recorded in the NHIRD were made on the basis of International Classification of Diseases, Ninth Revision, Clinical Modification (ICD-9-CM) codes^14,15^.

Before release, the data are anonymised. This therefore obviated the necessity of obtaining participants’ informed consent for this study. Because of the anonymity of the beneficiaries’ information, our executed study neither violated the participants’ privacy nor negatively influenced their welfare^13,15^. Chang Gung Memorial Hospital’s Institutional Review Board ratified our study (IRB No.: 201601249B1), with the executed study methods conforming to approved guidelines as well as regulations.

### Study cohort

NHIRD lists SLE under the “catastrophic illness” category. The Taiwanese government certifies SLE patients through a process that entails closely assessing medical records in addition to pathological and serological reports supplied by medical professionals^1,3^. Subsequently, the patients are included in the Registry for Catastrophic Illness Patients (RFCIP) and are entitled to substantial discounts on medical expenses. This confirms the reliability and accuracy of SLE diagnosis received by the enrolled patients.

**Figure 1:**
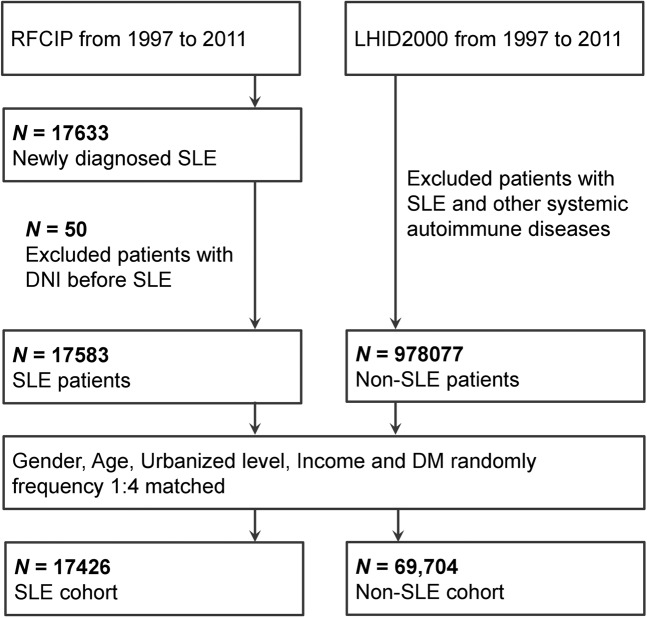
Study and comparison cohort enrolment process. Abbreviations: SLE, systemic lupus erythematosus; RFCIP, Registry for Catastrophic Illness Patients; LHID2000, Longitudinal Health Insurance Database 2000; DM, diabetes mellitus; DNI, deep neck infection; ICD-9, International Classification of Diseases, Ninth Revision.

From the Taiwan RFCIP, we retrieved data of patients newly diagnosed as having SLE for the period from January 1997 to December 2011 (Fig. 1). To make sure that we had a 2-year follow-up period at minimum, we disregarded patients receiving an SLE diagnoses after 2011. We employed ICD-9-CM code 710 associated with SLE, as defined in RFCIP^16^. We also disregarded in this study patients receiving a DNI diagnosis prior to receiving their SLE diagnosis.

### Comparison cohort

From the Longitudinal Health Insurance Database 2000 (LHID2000), which is an NHIRD subset consisting of data of 1,000,000 insurance beneficiaries randomly selected from all beneficiaries in 2000, we collected data to establish our comparison cohort^15,17^. The National Health Research Institutes has published reports^1,3,15^ revealing no significant disparity in healthcare costs or sex or age distribution between the sample group derived from LHID2000 and all beneficiaries in the NHIRD. Several population-based studies have employed LHID2000^1,3,12–15^. Here, our established comparison cohort contained patients without SLE or other systemic autoimmune diseases [based on these ICD-9-CM codes: 443.1 (Buerger disease), 446.0 (polyarteritis nodosa), 446.2 (hypersensitivity angiitis), 446.5 (giant cell arteritis), 446.7 (Takayasu disease), 696.0 (psoriatic arthropathy), 696.1 (psoriasis), 710.4 (polymyositis), and 714.0–714.4 (rheumatoid arthritis)].

### Matching process

With four randomly selected non-SLE patients from LHID2000, we matched each patient with SLE according to diabetes mellitus (DM), income level, age, urbanisation level, and sex. For our included SLE patients, we considered the index date to be the date on which they were registered in the RFCIP, and for the matched non-SLE patients, we considered it to be the same date as that of their matched SLE patient.

### Main outcome: DNI incidence

Here, DNI incidence was the main outcome. We defined such incidence to be any hospitalisation for the infections outlined as follows: parapharyngeal abscess (ICD-9-CM code: 478.22), cellulitis and abscess of oral soft tissues (Ludwig angina; ICD-9-CM code: 528.3), cellulitis and abscess of neck (ICD-9-CM code: 682.1), and retropharyngeal abscess (ICD-9-CM code: 478.24)^1,3,5^. We defined the follow-up period as that spanning from the index date to the DNI diagnosis date or date of death or 31 December 2013.

### Comorbidities

Several diseases that can lead to an immunocompromised status can be DNI risk factors; these include chronic kidney disease (CKD), DM, and liver cirrhosis (LC)^1,3,4,18^. In addition, tonsillectomy is confirmed a DNI risk factor^19^. Therefore, both our cohorts were evaluated for these comorbidities. The comorbidities were included if their ICD-9-CM codes were noted in the claims data at least once for inpatients or at least thrice for outpatients; thus, following ICD-9-CM codes were included: DM (250), hypertension (HTN; 401–405), CKD (403, 404, 585, and 586), cerebrovascular accident (CVA; 430–438), LC (571.2 and 571.5–571.6), and coronary artery disease (CAD; 410–414)^1,3,13–15,17,20^.

### Therapeutic modalities

We subcategorized our included patients into the following groups according to the therapeutic modalities applied to them: “surgical” group, comprising patients undergoing surgical intervention; and “nonsurgical” group, comprising patients receiving antibiotic or abscess aspiration without surgery^1,3^.

### Prognosis evaluation

We analysed prognosis using the hospitalisation duration, intensive care unit (ICU) admission, tracheostomy performance, and mediastinal complications—all defined in accordance with ICD-9-CM codes for mediastinitis (510, 513, and 519.2) or on the basis of whether patients received mediastinal surgery during hospitalisation^1,3^. For both cohorts, we investigated mortality (death in the course of DNI treatment) and mortality related to mediastinitis (death in the course of DNI treatment along with a mediastinitis diagnosis)^1,3^.

### Association of DNI occurrence with steroid dose for SLE therapy

SLE is typically treated using steroids with and without immunomodulators^6^. During the acute or unstable phase of SLE, high doses of steroids are administered, whereas in its chronic or stable stage, low doses of steroids are prescribed to control disease activities and prevent related complications. Chang *et al*.^6^ reported an average dose of 3 mg/day of prednisolone or equivalent to be the threshold for defining high or low doses of steroids in SLE patients. Therefore, we divided our SLE patients into two groups based on their average daily dose of prednisolone or equivalent during the period spanning between the index date and the conclusion of their follow-up.

### Statistical analysis

To compare the SLE and non-SLE cohorts’ demographic characteristics and comorbidities, we executed the unpaired Student *t* and Pearson chi-square tests for continuous and categorical variables, respectively. In the univariate analysis executed in this study, control variables constituted the covariates, namely urbanisation level, sex, comorbidities (CAD, LC, HTN, DM, CVA, and CKD), age, and income level. In the multivariate analysis implemented in the study, we included only variables whose *p* values were determined to be <0.1. Through the execution of Kaplan–Meier analysis, we could assess the cumulative incidence in the two cohorts; determined differences through the use of a two-tailed log-rank test. Next, we measured the hazard ratios (HRs) along with the corresponding 95% confidence intervals (CIs) of the incidence of DNI in the SLE and non-SLE cohorts by using multivariable Cox proportional hazard regression models. Moreover, we executed subgroup analysis and sensitivity testing to assess the stability of the SLE effect on DNI. We executed the entirety of the analysis procedures on SAS (version 9.4; SAS Institute, Cary, NC, USA), with statistical significance level being set to *p* < 0.05.

## Results

The sociodemographic characteristics, DNI incidence, and comorbidities in the SLE and non-SLE cohorts are presented in Table 1. In total, 17,426 SLE cases and 69,704 comparison cases were included. We observed the non-SLE cohort to exhibit a significantly lower CAD, LC, HTN, CVA, and CKD prevalence than did the SLE cohort (Table 1). Of all SLE and non-SLE patients, DNI was noted in 96 (incidence rate, 60 per 100,000 person-years; mean follow-up period, 9.18 ± 4.69 years) and 91 (incidence rate, 13.1 per 100,000 person-years; mean follow-up period, 10.0 ± 4.37 years), respectively. Thus, DNI incidence was determined to be significantly higher in the SLE cohort (*p* < 0.001). The mean duration from SLE diagnosis to DNI occurrence was 5.4 ± 4.2 years. Table 2 presents overall DNI incidence and that during <1, 1–5, and >5 years of follow-up in the two cohorts. The overall individual incidence rate ratio (IRR) of SLE patients compared with non-SLE patients was 4.59 (95% CI: 3.45–6.12); moreover, at <1, 1–5, and >5 years of follow-up, IRRs (95% CIs) were 7.66 (3.25–18.07), 6.06 (3.74–9.82), and 3.35 (2.24–5.02), respectively (all *p* < 0.001).

**Table 1:** Demographics and characteristics of SLE and non-SLE cohorts.

Characteristic	SLE		Non-SLE		*p*-value
	N	%	N	%	
Total	17426		69704		
Sex					1.000^*^
Male	2154	12.4	8616	12.4	
Female	15272	87.6	61088	87.6	
Age (years)					1.000^*^
<25	5056	29.0	20224	29.0	
≥25	12370	71.0	49480	71.0	
Urbanisation level					1.000^*^
1 (City)	5171	29.7	20684	29.7	
2	7822	44.9	31288	44.9	
3	2559	14.7	10236	14.7	
4 (Village)	1874	10.8	7496	10.8	
Income^†^					1.000^*^
0	7779	44.6	31116	44.6	
1–15840	2527	14.5	10108	14.5	
15841–25000	5141	29.5	20564	29.5	
≥25001	1979	11.4	7916	11.4	
Comorbidities					
DM	1465	8.4	5860	8.4	1.000^#^
HTN	6362	36.5	12318	17.7	<0.001^#^
CKD	2542	14.6	1486	2.1	<0.001^#^
LC	528	3.0	683	1.0	<0.001^#^
CAD	1970	11.3	5221	7.5	<0.001^#^
CVA	2034	11.7	3618	5.2	<0.001^#^
Tonsillectomy	4	0.02	22	0.03	0.556^#^
Outcome					
DNI	96	0.6	91	0.1	<0.001

Abbreviations: SLE, systemic lupus erythematosus; DM, diabetes mellitus; HTN, hypertension; CKD, chronic kidney disease; LC, liver cirrhosis; CAD, coronary artery disease; CVA, cerebrovascular accident; DNI, deep neck infection.

^†^NTD, per month.

*Pearson chi-squared tests.

^#^Student *t* test.

**Table 2:** Overall DNI incidence and that during <1, 1–5, and >5 years of follow-up in SLE and non-SLE cohorts.

Follow-up	SLE				Non-SLE				IRR	95% CI	*p*-value
	N	DNI	PY	Rate	N	DNI	PY	Rate			
Overall	17426	96	160028.8	60.0	69704	91	696808.3	13.1	4.59	(3.45–6.12)	<0.001
<1	17426	15	17047.8	88.0	69704	8	69642.0	11.5	7.66	(3.25–18.07)	<0.001
1–5	16801	40	61318.6	65.2	69568	28	260076.2	10.8	6.06	(3.74–9.82)	<0.001
>5	13250	41	81662.4	50.2	57400	55	367090.1	15.0	3.35	(2.24–5.02)	<0.001

Abbreviations: PY: person-years; IRR: incidence rate ratio; CI: confidence interval.

**Figure 2:**
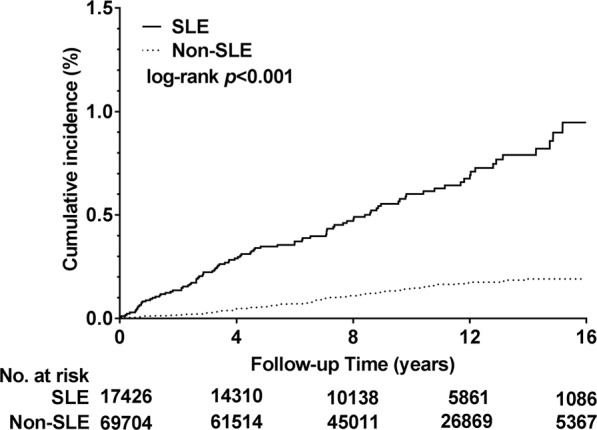
Cumulative DNI incidence in SLE and non-SLE cohorts by Kaplan–Meier analysis over the observation period (1997–2013). Log-rank analysis results indicated that DNI incidence was significantly higher in the SLE cohort than in the non-SLE cohort (*p* < 0.001).

The results obtained from the Kaplan–Meier analysis executed in this study revealed the cumulative DNI incidence over the observation period (1997–2013) in the two cohorts. Moreover, log-rank analysis results indicated the SLE cohort to exhibit a significantly higher DNI incidence (*p* < 0.001; Fig. 2). According to the results of the Cox proportional hazard regression executed in this study, the crude HRs and HRs adjusted for urbanisation level, sex, DM, income level, and age were obtained for both groups. DNI risk was 4.7-fold (95% CI: 3.50–6.32) greater in the SLE patients cohort when compared with the non-SLE cohort (*p* < 0.001; Table 3). The results obtained from the sensitivity analysis, in which a selected covariate was added to the main model, revealed that the effect of SLE on DNI was considerable and stable, and its significance was sustained in all subgroups, except for the LC, CKD, and CVA subgroups, according to the subgroup analysis results. Moreover, in the tonsillectomy subgroup, which was extremely small (n = 4 in study cohort and 22 in comparison cohort), DNI risk could not be calculated because no DNI occurrence was noted.

**Table 3:** Multivariable Cox proportional hazards regression analyses of association of DNI with the potential risk factors.

Variables	Adjusted HR	95% CI	*p*-value
Main model^*^	4.70	(3.50–6.32)	<0.001
***Additional covariates***^†^			
Main model + HTN	4.64	(3.42–6.31)	<0.001
Main model + CKD	4.84	(3.57–6.55)	<0.001
Main model + LC	5.10	(3.76–6.91)	<0.001
Main model + CAD	4.71	(3.51–6.34)	<0.001
Main model + CVA	4.66	(3.45–6.28)	<0.001
***Subgroup effects***^***‡***^			
Sex			
Male	3.68	(1.73–7.82)	0.001
Female	4.92	(3.56–6.79)	<0.001
Age (years)			
<25	5.35	(3.07–9.31)	<0.001
≥25	4.47	(3.15–6.34)	<0.001
DM			
Yes	2.39	(1.12–5.12)	0.025
No	5.34	(3.86–7.38)	<0.001
HTN			
Yes	2.77	(1.42–5.39)	0.003
No	4.97	(3.33–7.41)	<0.001
CKD			
Yes	1.41	(0.19–10.34)	0.733
No	3.80	(2.28–6.32)	<0.001
LC^§^			
Yes	—	—	—
No	5.07	(3.73–6.88)	<0.001
CAD			
Yes	6.08	(1.63–22.68)	0.007
No	4.73	(3.42–6.55)	<0.001
CVA			
Yes	3.04	(0.70–13.23)	0.139
No	4.80	(3.47–6.64)	<0.001

Abbreviations: HR, hazard ratio; CI, confidence interval.

^*^Main model adjusted for sex, age, urbanisation, income, and DM.

^†^Models adjusted for covariates in the main model and for every additional listed comorbidity.

^‡^Model is adjusted for sex, age, urbanisation, income, and comorbidities.

^§^For subgroup analysis under main model for patients with LC, the number of cases is insufficient for statistical analysis.

The therapeutic modalities for DNI employed in the two cohorts were antibiotics only or antibiotics plus abscess aspiration (nonsurgical group) or surgical drainage (surgical group); these modalities, along with other factors, including tracheostomy performance, hospitalisation duration, ICU admission, mediastinal complications, and mortality, are presented in Table 4. Regarding the proportion of treatment, SLE–DNI patients received less surgical intervention (SLE vs. non-SLE = 15.6% vs. 28.6%, *p* = 0.033). The between-group differences in tracheostomy performance (SLE vs. non-SLE = 3.1% vs. 3.3%, *p* = 0.947), hospitalisation duration (SLE vs. non-SLE = 12.86 ± 18.26 vs. 10.12 ± 22.17 days, *p* = 0.356), and ICU admission (SLE vs. non-SLE = 5.2% vs. 9.9%, *p* = 0.224) were all nonsignificant. The only two cases of mortality among all of the patients considered herein occurred in the non-SLE–DNI patients.

**Table 4:** Treatment, severity, and prognosis in SLE–DNI and non-SLE–DNI patients.

Characteristic	SLE–DNI		Non-SLE–DNI		*p*-value
	N	%	N	%	
**Total**	96		91		
**Therapy**					0.033^*^
Antibiotic ± Aspiration	81	84.4	65	71.4	
Surgery	15	15.6	26	28.6	
**Severity**					
Tracheostomy	3	3.1	3	3.3	0.947^#^
Hospitalisation^‡^	(12.86 ± 18.26)		(10.12 ± 22.17)		0.356^†^
ICU admission	5	5.2	9	9.9	0.224^#^
Mediastinitis	2	1.1	0	0.0	
**Prognosis**					
Mediastinitis-Mortality	0	0.0	0	0.0	
Mortality	0	0.0	2	2.2	

^*^Pearson chi-squared test.

^#^Fisher exact test.

^†^Student *t* test.

^‡^Mean ± standard deviation (days).

Abbreviations: ICU, intensive care unit.

**Figure 3:**
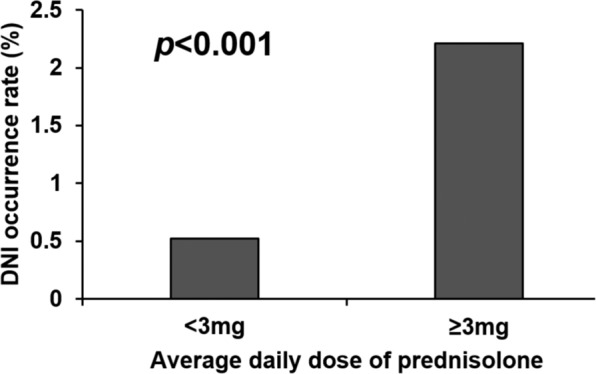
DNI incidence in SLE patients treated with high and low steroid doses. Average daily prednisolone or equivalent doses of >3 mg for SLE treatment increased DNI occurrence compared with lower steroid doses (*p* < 0.001).

By using the data presented in Fig. 3, we analysed incidence of DNI in SLE patients administered high (≥3 mg/day) or low (<3 mg/day) doses of steroids. Of the 183 patients administered high steroid doses, 6 developed DNIs (2.21%)—significantly higher than that in patients administered low steroid doses (90 in 17,243 patients, 0.52%; *p* < 0.001).

## Discussion

Our executed nationwide population-based study entailed the use of data from a real-world database. This is the first study to be executed on the influence exerted on DNI occurrence and prognosis by SLE. The derived results confirm SLE to be a definite DNI risk factor and DNI risk to be approximately five times higher in SLE patients than in non-SLE patients.

Studies have reported that SLE can increase infection and mortality risks, particularly in patients on a high steroid or immunosuppressant dose, with the respiratory tract being the most commonly involved, followed by the bloodstream, and bacteria being the most common causative agent, followed by viruses and fungi^21–23^. Moreover, infection rate is highest in the initial stages after SLE diagnosis, particularly the initial 5 years^22^. An SLE project and a large-scale case series revealed that infection accounted for the first cause of mortality within the initial 5 years after disease onset^24,25^. In the current study, DNI developing after SLE had a mean duration of 5.4 years. In addition, the IRRs for SLE–DNI compared with the comparison cohort over <1, 1–5 and >5 years of follow-up are presented in Table 2. IRR was highest for DNI diagnosis at <1 year of follow-up (7.66), followed by that at 1–5 (6.06) and >5 (3.35) years of follow-up.

Acquired deficiency of regulatory T cells for self-immunologic intolerance is thought to be an aspect of SLE pathogenesis^26,27^. Sjögren syndrome concomitant with SLE is common, and the immune defences of the oral cavity can be attenuated by xerostomia^28^. Therefore, oral infections, including periodontitis and tooth decay, are typically latent in SLE patients. In addition, steroids are the main medications administered in SLE therapy^29^, and they may cause immunosuppression, thus making patients susceptible to infection^7–9^, particularly patients administered high doses^6,9^. Based on the preceding evidence, the aforementioned factors could constitute the etiologies for the higher incidence of DNI in SLE patients.

Surgical debridement accounts for 20–80% of all therapies for DNI^1,3,4,30–34^. In our study, 15.6% of SLE–DNI patients and 28.6% of non-SLE–DNI patients received surgical treatment. The ratio of DNI patients who underwent surgical drainage is lower in this real-world study than the ratios in previous studies in which patients from tertiary medical centres were enrolled^4,11,32,34^. This study was based on a nationwide population-based database that includes data from primary to tertiary hospitals as well as those of patients with low DNI severity. This result may provide a complete spectrum of DNI treatment and prognosis and is consistent with the results of previous DNI-relevant population-based studies^1,3^. There were between-group differences in tracheostomy performance, hospitalisation duration, and ICU admission between the two cohorts, implying that DNI severity and prognosis in patients with SLE do not differ significantly from those in non-SLE–DNI patients.

In clinical applications, steroids may be administered to patients with acute or unstable SLE ether alone at high doses or in combination with immunosuppressants^6,9^. High doses of steroids, concomitant immunosuppressants, and high lupus activity would attenuate immune defences and engender more severe infection. We used the threshold given by Chang *et al*.^6^ (i.e. average daily dose = 3 mg of prednisolone or equivalent) to evaluate the incidence of DNI in SLE patients. The results indicate that high average daily doses of steroids (≥3 mg of prednisolone or equivalent) increased infection and mortality rates compared with low average daily doses of steroids. Danza *et al*.^9^ found that SLE patients treated with a daily dose of more than 7.5 mg of methylprednisolone were at an increased infection risk. In our study, SLE patients who were administered high doses of steroids exhibited significantly higher rates of DNI incidence (high dose: ≥3 mg/day vs. low dose: <3 mg/day = 2.21% vs. 0.52%, *p* < 0.001), thus corroborating previous findings^9^.

The strengths our study include the large sample size (17,426 patients with SLE, representing a nationwide spectrum) and long follow-up period (9.84 ± 4.43 years). However, the following are the few limitations of our population-based study: First, medical images such as computed tomography and magnetic resonance imaging were not available; hence, we could not confirm the extent of DNI and the size of the abscess. Second, blood- and pus-culture data could not be obtained from the NHIRD; we thus could not discern the definite bacterial spectra or drug sensitivities of our patients with DNI. Third, the definite causes of death could not be obtained from the NHIRD; therefore, as an alternative to DNI-specific mortality, we investigated 3-month mortality. Finally, because this retrospective study used claims data, several factors, including patients’ clinical presentation and treatment course, physical examination findings, and laboratory study results, could not be evaluated in detail. Additional studies elucidating the causal relationship of SLE with DNI as well as the related treatment outcomes are warranted.

## Conclusions

This real-world study is the first to investigate SLE and the incidence, treatment, and prognosis of DNI. Our findings strongly support the assertion that SLE is a DNI risk factor. Patients with SLE under high-dose steroid treatment demonstrated higher DNI incidence rates than did those under low-dose steroid treatment. Fewer SLE–DNI patients were subjected to surgical treatment compared with the non-SLE–DNI patients. Finally, the differences in mortality, tracheostomy performance, ICU admission, and hospitalisation duration between SLE–DNI and non-SLE–DNI patients were nonsignificant.

## Data Availability

The datasets generated or analysed in the current study can be accessed from the Taiwan National Health Insurance Research Database repository (https://nhird.nhri.org.tw/en/How_to_cite_us.html).
